# CMR evaluation in patients with high grade ventricular arrhythmias

**DOI:** 10.1186/1532-429X-11-S1-P205

**Published:** 2009-01-28

**Authors:** Antonio Bernardini, Agostino Meduri, Luigi Natale, Carlo Liguori, Riccardo Fenici, Donatella Brisinda, Lorenzo Bonomo

**Affiliations:** 1 "G. Mazzini" Hospital – ASL Teramo, Teramo, Italy; 2 Università Cattolica del Sacro Cuore – Policlinico A. Gemelli, Rome, Italy; 3 Università Cattolica del Sacro Cuore, Rome, Italy

**Keywords:** Ventricular Arrhythmia, Wall Motion Abnormality, Leave Bundle Branch Block, Right Bundle Branch Block, Premature Ventricular Complex

## Purpose

To assess by cardiac-MR the prevalence of myocardial alterations in arrhythmic patients.

## Materials and methods

43 patients with non ischemic ventricular arrhythmias. Premature ventricular complexes had left bundle branch block morphology (LBBB) in 29 cases, in 7 a right bundle branch block contour (RBBB) and 7 had polymorphic patterns(PV). US was negative in 78.4% of patients, while CMR was negative in only 13% of patients. Studies were performed on a 1.5 MR scanner with Cine sequences (Fastcard or FIESTA), bb-FSE and IR-prep FGRE 15 minutes after injection of 0.2 mmol/Kg of Gd-DTPA.

## Results

CMR found a high prevalence of morphological, signal intensity and functional myocardial abnormalities. RV dilatation was found in 85% of patients with PV arrhytmias, 48.3% of patients with LBBB morphology, 12.5% of patients with RBBB morphology. LV dilatation was present in 28.6%, 25% and 24.1% of patients with LBBB, PV and RBBB type arrhytmias respectively. RV wall motion abnormalities were identified in 50% and 36.7% of patients with PV and LBBB pattern respectively while LV motion abnormalities in 25% and 10.3%. Free wall RV signal/thickness abnormalities were found in 23.3% of patients; LV signal abnormalities were found in 11.6% of patients.

Seven patients underwent myocardial biopsy: 5 positive for myocarditis, 1 positive for ARVD, one had a negative biopsy.

## Conclusion

In patients with primary ventricular arrhythmias MR documented high prevalence (87%) of morphological, signal intensity and wall motion abnormalities even with negative echocardiogram.

